# What does it mean to conduct participatory research with Indigenous peoples? A lexical review

**DOI:** 10.1186/s12889-019-7494-6

**Published:** 2019-10-29

**Authors:** Ann Dadich, Loretta Moore, Valsamma Eapen

**Affiliations:** 1 School of Business, Western Sydney University, 169 Macquarie Street, Parramatta, NSW 2150 Australia; 2 1797 Locked Bag, Western Sydney University, 1797 Locked Bag, Penrith, NSW 2751 Australia; 3 Autism Spectrum Australia (Aspect), Building 1, Level 2, 14 Aquatic Drive, Frenchs Forest, NSW 2086 Australia; 4 Forestville, NSW 2087 Australia; 5 Academic Unit of Infant, Child, Adolescent Psychiatry South West Sydney, University of New South Wales, Sydney, Australia; 6 ICAMHS, L1 MHC, Liverpool Hospital, Elizabeth Street, Liverpool, NSW 2170 Australia; 7 University of New South Wales Sydney, Sydney, NSW 2052 Australia

**Keywords:** Indigenous research, Participatory methodologies, Lexical analysis, Knowledge translation

## Abstract

**Background:**

To better understand and promote public health, participatory research with Indigenous peoples represents recommended practice, worldwide. However, due to the different ways such research is referred to, described, and used, it is unclear what might (and might not) warrant the term when collaborating with Indigenous peoples. As such, this article expands conceptual understandings of participatory research with Indigenous peoples, across timelines and regions.

**Method:**

Following a systematic search of 29 academic databases in April 2018, a lexical analysis of the methods sections was conducted, which were sourced from 161 publications across 107 journals.

**Results:**

The active involvement of Indigenous peoples in research that is expressly participatory is limited across all project phases. This might be because the ways in which Indigenous peoples were involved throughout were not reported – however, it might also be because Indigenous peoples were not involved in all project phases. Furthermore, descriptions differ by study location and publication timeframe – notably, studies in the region of the Americas chiefly refer to pandemics, surveyors, and art; and those published in the last two decades have given primacy to artifacts of interest.

**Conclusions:**

Findings from this corpus of data suggest participatory research with Indigenous peoples is not always described across different project phases; furthermore, it differs according to study location and publication timeframe. This offers considerable opportunity to further this important research area via alternative methodologies that award primacy to Indigenous expertise and agency.

**Electronic supplementary material:**

The online version of this article (10.1186/s12889-019-7494-6) contains supplementary material, which is available to authorized users.

## Background

To redress the power imbalance in research with Indigenous peoples, participatory research represents recommended practice, worldwide [1]. This recommendation recognizes that, ‘Discrimination against… indigenous peoples… causes and magnifies poverty and ill-health’ [2]. Conventional, non-participatory approaches have largely failed to address health inequalities, with Indigenous peoples more likely to experience poor health, reduced quality of life, and premature death, relative to their non-Indigenous counterparts. Consider for instance, the ways in which some research approaches, steeped in colonialism, have subjugated Indigenous voices. As Indigenous scholar, Tuhiwai Smith [3], critiqued:

Research[ers] within late-modern and late-colonial conditions… enter communities armed with goodwill in their front pockets and patents in their back pockets… Research… *on* indigenous people is still justified by the ends rather than by the means… [Some] researchers… collect… beliefs systems and ideas about healing, about the universe, about relationships and the ways of organizing… The global hunt for new knowledges… brings new threats to indigenous communities… what counts as Western research… (1) allow[s] ‘us’ to characterize and classify societies into categories, (2) condense[s] complex images of other societies through a system of representation, (3) provide[s] a standard model of comparison, and (4) provide[s] criteria of evaluation against which other societies can be ranked… These are the procedures by which indigenous peoples and their societies were coded into the Western system of knowledge [3, original italics].

Research *on* Indigenous peoples differs from *with* Indigenous peoples, which requires ‘relational accountability’ [4, 5]. At best, research *on* Indigenous peoples yields findings of reduced validity and reliability [6]; at worst, it exacerbates the longstanding overrepresentation of Indigenous peoples who experience poor wellbeing [7, 8].

Although the value of participatory research with Indigenous peoples is not contested, it has varied understandings [9]. For instance, Windsor and colleagues [10] described ‘a 2 × 2 × 2 × 2 factorial design to engineer the most efficient, effective, and scalable version’ of a behavioral-health intervention, which was ‘Grounded in critical consciousness theory, community-based participatory research principles’. In essence, participatory research was used to design a cost-effective intervention for wide-spread use, by ‘ensur[ing]… that research questions and procedures reflect[ed] the needs and priorities of the communities… [to] facilitate[e] uptake’. Yet another approach by Genuis and colleagues [11] reported on high-school students at a First Nation community school who were trained as co-researchers. Specifically, nine students were ‘recruited as project co-researchers’, incentivized through an ‘offer… [of] credit towards classroom assignments, as well as an opportunity to positively impact their community’. Following ‘5 training sessions with university investigators’, the co-researchers ‘conducted semi-structured… interviews’, which were primarily ‘analys[ed] by university researchers’. These two (of many other) examples demonstrate the different ways in which Indigenous peoples are involved in participatory research. Although variation within most methodologies might be expected [12], it is unclear what might (and might not) warrant the term when collaborating with Indigenous peoples [13].

Given increasing interest in such research [14], this article expands conceptual understandings of participatory research with Indigenous peoples, across timelines and regions. This was achieved via a lexical analysis of the methods sections of 161 publications, identified via a systematic review of academic databases. Aided by the software program – Leximancer – a lexical analysis involves the examination of a corpus of qualitative data to ascertain patterns in the ways in which words – and their associated phrases – travel together [15]. This is achieved via ranked lists of terms that commonly occur and co-occur, from which a thesaurus is built to delineate salient themes and the concepts, therein. This article commences with an overview of participatory research with Indigenous peoples. It then presents the findings from the aforesaid lexical analysis. The article then concludes with a discussion of key findings, and the associated implications.

### Participatory research with indigenous peoples

Participatory research with Indigenous peoples prizes partnership between individuals (and/or the groups they represent) who have a stake in the research, including (but not limited to) Indigenous peoples and researchers [6]. This partnership involves equal opportunities for engagement between different individuals (and/or the groups they represent) to pursue a common purpose by sharing and generating knowledge [16]. Accordingly, participatory research can range in scope and form – for instance, it can involve individuals who identify as Indigenous, and/or communities that identify as Indigenous. The latter involves partnerships between an Indigenous community and research agencies, and can range from, ‘being consultative through community-directed to community-controlled, where community groups exercise the highest expression of autonomy over research, assisted by research institutions’ [17]. Participatory research aims to democratize scholarship and knowledge by: relegating conventional understandings of expertise and evidence; ‘shift… the balance of control towards those being researched’ [18]; and reposition scholars as participants of a process in which they listen, learn, and offer service [19–21]. In effect, it is the intersection of skillsets to enhance the translation of the outcomes associated with the partnership into policy and/or practice. While conventional research typically prioritizes professional and institutional interests, disciplinary conventions, and codified-forms of evidence, participatory research with Indigenous peoples prioritizes culture and community [19].

Although participatory research with Indigenous peoples can range in scope and form, it is premised on some key principles [22–24]. According to the World Health Organization [1], both research institutions and Indigenous peoples are to ‘enter into a research relationship as equal partners’, whereby both parties develop a proposal and endorse an agreement – however, the research should only proceed if its focus and processes align with the priorities and needs of Indigenous peoples. This is not to suggest that translating these principles into practice is always straightforward – for instance, Morton Ninomiya and Pollock [25] described accountability tensions when research teams have a responsibility to ‘the “hands off” community leaders and stakeholders involved in… research and… the “hands on” academic world, full of rules and regulations’. Nevertheless, there are many international exemplars in which such tensions have been respectfully managed with considerable success – consider for instance, a large-scale, community-based participatory research project to address the high-rate of tobacco smoking among Aboriginal and Torres Strait Islander peoples across Australia [17, 26, 27]. The principles espoused by the World Health Organization [1] were adapted for the purpose of a reporting framework to clearly articulate what each of the seven project phases would involve and how each would be assessed to ultimately democratize the participation of the partners. This and other international studies make a strong public health case for participatory research with Indigenous peoples [28–32].

In addition to being respectful, such research can help to address intractable public health problems, where conventional epidemiological approaches have achieved limited success [33]. For instance, following their ‘comprehensive literature review’, Bath and Wakerman [20] found some evidence that participatory approaches are associated with improved health outcomes within Indigenous communities. Similarly, in their systematic review on community development projects in Australian Indigenous communities, Snijder and colleagues [34] identified two studies that reported statistically significant outcomes. Although there is a dearth of empirically-robust research (as conventionally-defined), available evidence suggests that participatory research with Indigenous peoples holds potential and is worthy of future scholarship.

The aim of this article is to determine what it means to conduct participatory research with Indigenous peoples. Specifically, it examines how such research was described. This was achieved via a lexical analysis of relevant publications. Given the aforesaid aim of this article, all publications that are expressly participatory were considered. Rather than appraise the quality of studies with reference to an established research standard, this approach was deemed appropriate because of the varied understandings of such research with Indigenous peoples [9]. As such, the aim of this article is not to assess research quality – but rather, to investigate how research that is expressly participatory was described. Details of this lexical review are described as follows.

## Methods

### Search strategies

**Fig. 1:**
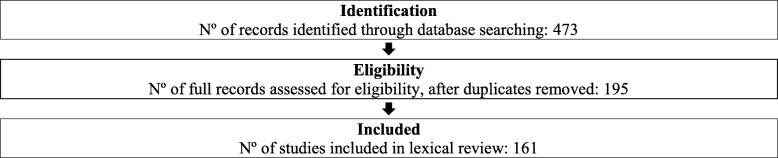
Flow Diagram of Study Selection [adaption of PRISMA, 36]

In April 2018, search strategies were deployed across 29 purposely-selected academic databases to identify all publications on participatory research with Indigenous peoples. Guided by previous research [35], the search strategies encompassed euphemisms for Indigenous (19 terms) and participatory (12 terms) within the publication title. Although potentially limiting, more inclusive search strategies largely served to identify irrelevant publications. The breadth of these publications might be partly due to multifaceted nature of both Indigeneity and participatory research. As such, a focused search strategy was used. Publications were included in this review if they met all three of the following criteria: (1) it represented a research publication, rather than a letter, a commentary, or an editorial, to ensure the inclusion of empirical research; (2) it did not represent a systematic, narrative, or literature review or meta-analysis, given the limited methodological detail typically reported from the publications that are included within such reviews; and (3) it was published in the English language. Of the 473 publications identified, 161 met these three criteria (see Fig. 1 and Additional file 1). This was determined by one author and cross-checked by another for accuracy. Discrepancies in this process were reconciled through consensus. The methods section from each publication was then extracted for a lexical analysis. Collectively sourced from 107 journals, the earliest publication was published in 1970, and the latest, in 2018.

### Lexical analysis

The lexical analysis was aided by Leximancer – data-mining software that uses Bayesian reasoning to detect key concepts and reveal their relationships [37]. By identifying frequently occurring and co-occurring words, Leximancer visually maps **concepts** that reflect **topics within the text** [38]. The maps convey ‘the main concepts in the text and their relative importance; the strengths of links between concepts (how often they co-occur); and similarities in contexts where links occur’ [39]. **Clusters of concepts** within a map – known as **themes** – suggest contextual similarity [40]. Themes are color-coded to signify those that are (and are not) important, whereby the ‘most important theme appears in red, and the next hottest in orange, and so on according to the colour wheel’ [41]. Further detail on Leximancer can be sourced from previous publications [42, 43].

For four key reasons, Leximancer was purposely selected to aid this review. First, it can offer a ‘helicopter’ view of a substantial body of qualitative data, illustratively portraying relationships and patterns between representative themes and concepts [44]. Second, as a form of computer assisted qualitative data analysis software (CAQDAS), it offers a systematic, logical, and an efficient method to ‘text mine’, allowing the researcher to interactively connect themes and concepts with the data. As Hyndman and Pill [45] noted:

The advantage of Leximancer is that it extracts a populated list from the text document that displays the weighted term classifications and connections between key words. From this list it creates concept maps that illustrate the level of connections between key words in the text being analysed… In other words, the software processes the level of relationship between concepts and the rate at which concepts and the significantly related terms appear close to each other within the text.

Third, unlike alternative approaches when systematically analyzing qualitative data – like the oft-cited use of thematic analysis [46–48] – Leximancer can help to reveal, and make sense of different findings [49]. Given its capacity to offer an ‘unsupervised’ view of the data [50], it can facilitate ‘broader opportunities for interrogating the text’ [51] by grounding the analysis in the voice of the authors of the data. This is not to suggest the limited value of alternative approaches – but rather, Leximancer can direct researcher attention to the unexpected (as well as what might be expected). As Smith [52] observed:

The meaning contained within any data set is very much dependent on the way you interpret it. Not only do you need to carefully decide what things to measure, but you need to understand what the analysis method is trying to achieve, and finally, you need to understand what this result actually tells you about your world… the only value to be obtained from textual data is a more accurate understanding of the way the authors of the data viewed some aspect of their world… if we assume that the task is for the analyst to understand the human meanings contained with their text data, the role of software such a Leximancer is… to let the data generate a transparent model which can be interpreted by the analyst, so that this person may efficiently conduct a sense making examination of conceivably vast amounts of text.

Fourth, although Leximancer has been used to systematically review literature in other fields – including (but not limited to) infection control [43], knowledge management [53], marketing [54], nursing [55], and physical education [45] – it is yet to be used to ‘text mine’ literature on participatory research with Indigenous peoples. Leximancer was therefore used because it was fit-for-purpose, helping to address the aim of this article and ensure the unexpected would be balanced with the expected.

Leximancer was used in two steps. First, once the methods section from each publication was collated, the ‘discovery’ mode was used to, ‘see what concepts were automatically generated by Leximancer without intervention’ [56]. Illustrating the automatically-generated relationships within the text, in the first instance, helps to ‘create learning and understanding’ [57] and identify ways to make sense of these relationships. Second, for comparative value, each publication was associated with two tags. Tagging helps to compare the conceptual content of different data [58]. To determine whether (and how) study location influences the ways participatory research with Indigenous peoples is described, each publication was tagged according to one of six regional groupings, as defined by the World Health Organization [59]. To determine whether (and how) time influences the ways participatory research with Indigenous peoples is described, each publication was tagged according to one of four timeframes – namely: ‘Seventies and Eighties’ (given that only one publication was published in 1970); ‘Nineties’; ‘Noughties’; and ‘Tensies’ (reflecting accepted vernacular). Once tagged, and guided by previous research [39], thirty concepts were profiled within each concept map to avoid diluting the focus of each map. To identify differences between locations and timeframes, the thirty concepts were profiled using the themed discovery setting, ‘Concepts in EACH’, to ‘discover concepts that distinguish… categories from one another’ [41]. For succinctness, attention is awarded to the word-like concept (rather than pronouns) that is most pertinent to each tag, as indicated by the likelihood percentage. Calculated by Leximancer, the likelihood percentage denotes the proportion of text segments that is shared by a tag concept and another concept, thus providing both directions of conditional probability [60].

## Results

**Fig. 2:**
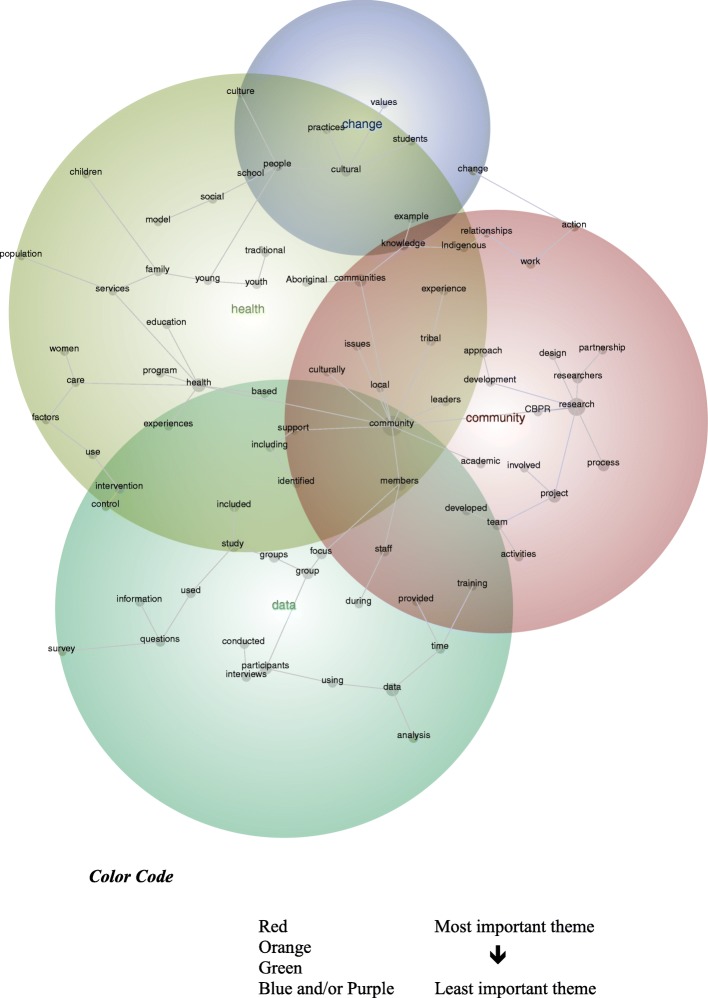
Discovery Mode Concept Map (visible concepts: 100%, theme size: 60%)

The discovery mode concept map reveals four themes – namely: *community*, *data*, *health*, and *change* (see Fig. 2). These highlight the key clusters of concepts – or topics – represented within the text. Theme position illustrates the relationships between the themes. Consider the prominence of *community*, which appears in red and overlaps with the less-prominent themes, particularly *data*. This suggests that when the publications refer to *community* (and the concepts therein), they are inclined to refer to *data* (and the concepts therein):

The team comprised health services researchers, physicians, Indigenous researchers, social scientists, data analysts, nurses, a community development specialist, and community-based experts and participants such as FNs [First Nations] Elders, health care workers and community members [61].

Given the focus of this review and the purposeful analysis of the methods sections, the absence of a concept that explicitly denotes *participatory*, *participation*, or *participate* is curious. Although the concept, *involved*, is evident within the salient theme, *community*, it is most-likely to be connected to the concept, *project*. This suggests that when the publications refer to being *involved*, they are inclined to refer to a *project*:

As well, in Saskatchewan, Sage, another participant described why she became involved in this PAR project. She said: I joined (this research project) because I know that I’ll get my word out [62].

Although this finding might appear intuitive, the concept, *involved*, is dissociated from specific project phases. Consider its distance from *intervention*, *control*, *survey*, *conducted*, *interviews*, and *analysis*. Furthermore, these are distanced from germane concepts like, *Indigenous* and *Aboriginal*. This suggests when the publications mention *involved*, *Indigenous*, or *Aboriginal* they are disinclined to refer to *intervention*, *control*, *survey*, *conducted*, *interviews*, and *analysis*. This is not to suggest that the publications do not describe how Indigenous peoples were actively involved in the collection or analysis of data – but rather, the methods, as presented in this corpus of data, suggest the former concepts (and the words within their thesaurus) seldom travel with the latter concepts (and the words within their thesaurus):

The Family Spirit intervention was staffed to provide regular on-site supervision, weekly cross-site conference calls and quarterly site visits. A policy and procedures manual guides implementation of the curriculum and gives home visitors flexibility to address mothers’ and families’ scheduling needs [63].

Perhaps the most explicit reference to participatory research is *CBPR* (community-based participatory research). Although positioned between *community* and *research*, it is in closer proximity to the latter. As such, *CBPR*, as described within the methods sections, is inclined to travel with words that denote *research*, rather than be equidistant from words that denote *community*:

A Community-Based Participatory Research (CBPR) framework was used to develop a qualitative study around young Indigenous people’s sexual health. Our participatory approach… involved a range of strategies to ensure the project was a genuine collaboration between university researchers and Indigenous community members, and in particular that young Indigenous people were actively involved throughout [64].

Given the publications explicitly focus on participatory approaches with Indigenous peoples, it is encouraging to observe *research* in close proximity to *process*, *involved*, *development*, *design*, and *partnership*. This suggests the methods sections speak of processual or progressive scholarship. In addition to the concept map and the exemplary excerpts, this is supported by the absence of concepts that denote outputs and deliverables:

Key terms and significant issues for data analysis were identified through a collective contribution process by the elders, leaders, knowledge-holders and youths during a subsequent traditional sharing circle. Participants wanted to be sure their needs and dreams were included in the draft findings, so that this research would have an impact on policy level and speak on behalf of them [65].

Another curious finding is the salience of *health* as a theme – this is because the search strategy was devoid of the term, health. Furthermore, of the academic databases searched, 15 were not (mental) health-specific. This suggests that wellbeing is a prominent focus in participatory research with Indigenous peoples, as presented in these systematically-identified publications:

People recognize that these diseases are transmitted by insects, which they call shidru (Triatominae, kissing bugs) for Chagas and shirakbina (Anopheles, mosquitoes) for malaria. Local health services consist of an infirmary attended by a nurse, and the nearest healthcare center requires 2 h of travel by river [66].

As illustrated by the grey spanning tree, *health* is most-likely to be connected with the concepts, *program*, *based*, *experiences*, *education*, *care*, *services*, and *community*. The spanning tree portrays, ‘the most-likely connections between concepts (like a road map of highways), but there are other (less-strong) connections between concepts (like backstreets)’ [58]. This finding is noteworthy for two key reasons. First, these connections speak of initiatives to intercede in, and/or affect wellbeing. This extends to the concept, *experiences*:

We explained that the data collected at these initial encounters would then inform the semi-structured interviews intended to further explore the young people’s understandings and experiences of health in the hopes that this would lead to a youth-led project [67].

Second, as per the preceding excerpt, references to these initiatives travel with references to particular cohorts – notably, *youth*, *women*, *family*, *students*, and *children*. These demonstrate the research priorities within this corpus of data. Consider the concept, *youth*, and its proximity to *traditional* and *Aboriginal*. This illustrates the connectedness between discourse pertaining to *youth* and Indigenous peoples and customs:

The focus group began with a welcome, introductions and an Inuk elder ceremonially lighting the *qulliq*, a traditional oil lamp. Apart from introductory and concluding activities, there were four main segments: understanding violence, coping with violence, preventing violence, and what Inuit youth should know about violence [68, original italics].

Although reference to the aforesaid cohorts is noteworthy, so too is the absence of expressed reference to others. In this regard, the concepts automatically generated by Leximancer did not include references to (or euphemisms for) men, the elderly, infants, people with a disability, or people who identify as lesbian, gay, bisexual, trans, and/or intersex, among others. This is not to suggest the publications ignored these cohorts, but rather, they did not feature prominently in the methods sections of these publications, all of which pertained to participatory research with Indigenous peoples.

In the context of publications that expressly focused on participatory research with Indigenous peoples, there is a curious divide between the themes, *data* and *change*. The concept map suggests that discourse pertaining to *participants*, *study*, *focus*, *groups*, *analysis*, *questions*, *interviews*, *intervention*, and *survey*, is not well-connected with that pertaining to *practices*, *values*, and *culture*:

This pedagogy is appropriate for Samoans since they have an oral tradition that values collective decision-making, experiential education, trust building, and interpersonal interactions [69].

Although it is not the purpose of this article to hypothesize reasons for this divide, this finding does not portray discourse pertaining to conventional demonstrations of research – including the collection and analysis of data – as inextricably connected to that pertaining to cultural values and practices. But rather, they appear disconnected.

**Fig. 3:**
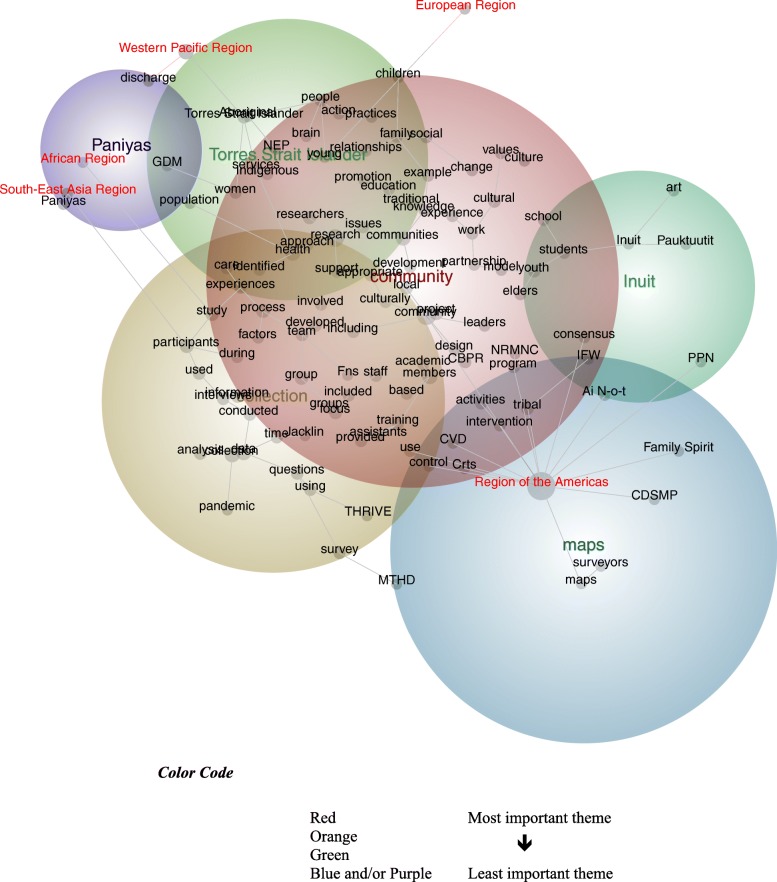
Concept Map tagged by Regional Groupings (visible concepts: 100%, theme size: 60%)

The 161 publications reported studies that were conducted across at least 16 nations, with one publication encompassing ‘communities from Siberia to Norway’. These publications represented five (of six) regional groupings, the exception being the Eastern Mediterranean region. Although the concept map is seemingly busy, this was necessary to ensure all five groupings are represented. This helpful comparison suggests study location influences the ways participatory research with Indigenous peoples is described, with variation between studies conducted within the region of the Americas, and those conducted elsewhere (see Fig. 3). Studies conducted within this region refer chiefly to the concepts, *pandemic* (100%), *surveyors* (100%), and *art* (100%):

to understand the barriers participants faced and suggested improvements for the pandemic response, the interview questions were based on the aspects of a health sector pandemic response outlined in academic literature [70, Region of the Americas].

Conversely, those conducted within the remaining four regions feature discourse pertaining to: *discharge* processes (Western Pacific Region: 100%); the *young* (European Region: 17%); *practices* (South-East Asia Region: 17%; African Region: 5%); and *care* (African Region: 5%). These nuances reveal two notable findings. First, there are shared interests among the studies conducted in the South-East Asia and African Regions, with reference to personal, social, and organizational practices:

Hygienic practices common in Kerala are not universally adopted; over a quarter of the households do not systematically boil their drinking water. Their health needs are great [71, South-East Asia Region].

Second, studies conducted in the Western Pacific Region are strongly connected with discourse on non-Indigenous healthcare conventions. These include the admission and release of patients from health services, and the artifacts accrued to codify patient care:

First, client details were hand-written into a service admission book upon intake and discharge. Data collected included: demographics; referral type; and service utilization characteristics [72, Western Pacific Region].

**Fig. 4:**
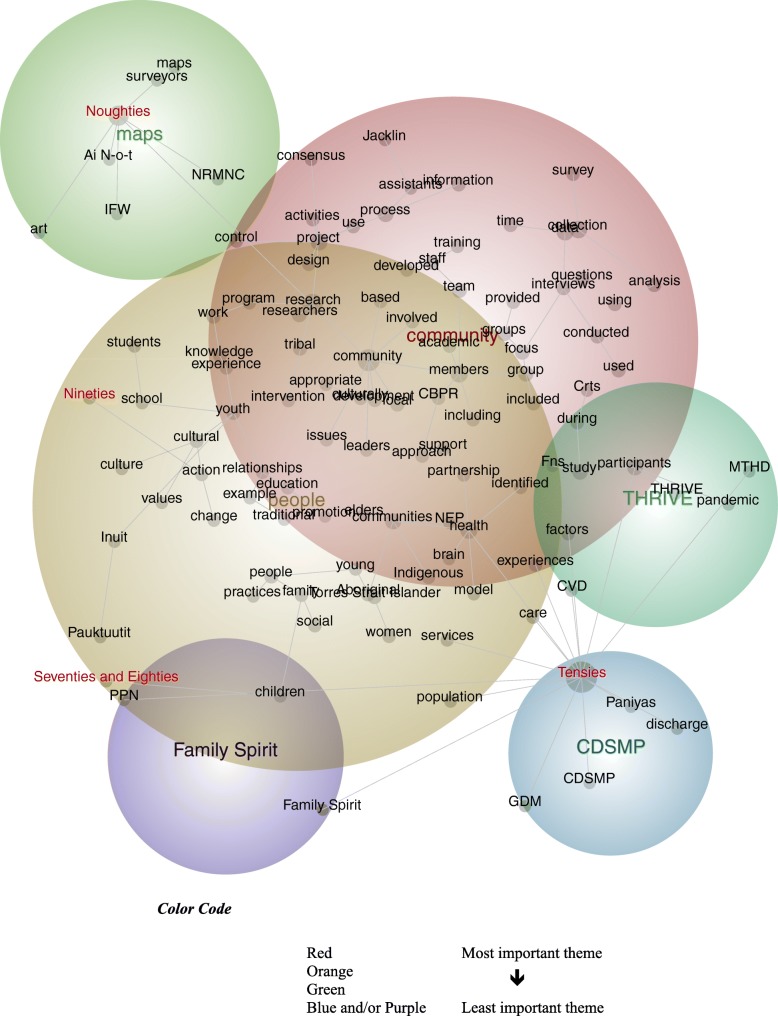
Concept Map tagged by Timeframes (visible concepts: 100%, theme size: 60%)

The 161 publications reported studies across the seventies and eighties (*n* = 2), the nineties (*n* = 6), the noughties (*n* = 43), and the tensies (*n* = 110). The centrality of the themes, *community* and *people*, suggest their salience across the decades (see Fig. 4). Although the concept map is heavily populated with concepts, this was necessary to ensure all four periods are represented. However, the likelihood percentages reveal key differences between these periods. Specifically, unlike more recent publications, those published the earliest are most-likely to be associated with discourse regarding *children* (Seventies and Eighties: 9%) and *action* (Nineties: 9%) – while those published during the last and current decades are strongly associated with discourse pertaining to *surveyors*, *art* (Noughties: 100%), and *discharge* (Tensies: 100%). These findings reveal a pattern in the ways participatory research with Indigenous peoples are described. While the methods sections of earlier publications present language about particular cohorts and change efforts to address identified issues, those of more recent publications award primacy to artifacts that denote areas of interest, including culture, the terrain, and service-use:

Hospital representatives reported that they were receiving fewer requests from community health centres for ‘missing’ discharge summaries and that the content of the discharge summaries had improved [73, Tensies].

## Discussion

The importance of participatory research with Indigenous peoples has international recognition. Yet there are discrepancies in the ways such research is conducted and reported. Participatory research with Indigenous peoples can help to improve research quality and optimize the relevance of associated outcomes for Indigenous peoples [20, 34]. It is important for researchers to reflect on how they engage with Indigenous peoples, given limited progress to redress longstanding health inequalities [74, 75].

The key finding from this lexical review is that publications that explicitly pertain to participatory research with Indigenous peoples do not always demonstrate Indigenous participation across different project phases. For instance, discourse regarding Indigenous peoples is distanced from that regarding the collection and analysis of data, and the reporting of the associated findings. Furthermore, the ways the research is collectively described suggests a disconnect between research and cultural values and practices. Although it is beyond the scope of this review to account for these findings, it is possible that although the studies were participatory, the ways in which Indigenous peoples were involved throughout were not reported – however, it might also be because Indigenous peoples were not involved in all project phases.

An examination of participatory research with Indigenous peoples across regions and decades reveals key differences. For instance, studies conducted within the Americas allude to pandemics, surveyors, and art; while those conducted within the Western Pacific region feature Western healthcare processes – this demonstrates differences in the focus of studies within each region. Over time, there have been considerably more publications reporting participatory research with Indigenous peoples – yet these descriptions have changed over time. While early publications consider particular cohorts of Indigenous peoples and change efforts to address the issues they experience, later publications demonstrate an interest in codified-forms of culture, the terrain, and service-use.

Collectively, these findings suggest that, across the globe and over time, participatory research with Indigenous peoples is understood, conducted, and described in disparate ways. Although disparity can optimize inclusiveness, it can be problematic for (at least) two reasons. First, it potentially dilutes scholarship, stymies the development of innovative solutions, and compromises theory-development – this is because researchers and Indigenous peoples engage with, and among each other without shared understandings. Second, research with Indigenous peoples might be inappropriately labelled as participatory and exacerbate longstanding inequalities [7].

Although the findings from this lexical review are illustrative, three limitations warrant mention. First, given the disparate ways in which participatory research and Indigenous peoples are described, it is possible that some relevant publications were obscured by the indexing systems used by the academic databases that were searched. Despite the comprehensiveness of each database, the terms that were searched are referred to, and defined in disparate ways. Second, because participatory research with Indigenous peoples is understood in different ways, the accounts reported in the publications could not be verified. Third, Leximancer regulates researchers’ analysis – although this can strengthen qualitative research [48], the use of alternative approaches, like (but not limited to) thematic analysis [45–47], might yield different findings.

Despite the aforesaid limitations, the key finding from this lexical review has implications for scholars, practitioners, and Indigenous peoples. For scholars, given the importance of impact, this review would suggest that there is much scope and opportunity to actively engage Indigenous peoples in all research phases to improve public health initiatives and redress longstanding health inequalities. Several methodologies have a demonstrated capacity to enhance engagement – consider for instance, citizen social science [76] and video reflexive ethnography (VRE, [77, 78]). Informed by both crowdsourcing and citizen science, citizen social science involves avocational researchers who examine social phenomena by collecting and analyzing data, disseminating the associated findings, and translating these into practice. In the context of participatory research with Indigenous peoples, citizen social science might involve inviting Indigenous peoples to collect, access, and/or critique practices that influence public health; share insights and experiences; identify knowledges and conditions that shape the translation of preferred practices into different contexts; and co-design resources (sensu *lato*) to promote public health outcomes.

VRE purposely harnesses the expertise of individuals who are typically relegated to the position of research subjects – like Indigenous peoples. Specifically, non-academic researchers are invited to collaborate as co-researchers by: featuring in and/or gathering video-recordings; analyzing the recordings; and understanding practices and experiences [79].

For practitioners, the findings provoke potentially challenging questions about how they conduct research and/or quality improvement exercises, and whether current practices serve to reinforce health inequalities [7]. For Indigenous peoples, given the seeming importance of their involvement in, and research about healthcare [80], these findings demonstrate the relative absence of participatory research. This then is a call to Indigenous peoples to hold scholars and practitioners to account by challenging, if not pushing the agenda of academic institutions, health services (sensu *lato*), and the governments that fund them.

## Conclusions

This lexical review suggests the active involvement of Indigenous peoples in research that is expressly participatory is limited across all project phases. Notwithstanding opportunities to engage Indigenous peoples in a ‘project’, there is limited clarity regarding their involvement in the collection and analysis of data, and the reporting of the findings. This suggests the expertise and skills of Indigenous peoples are not always harnessed. With exceptions [11, 81], Indigenous connections to research – as depicted in this corpus of data – was sometimes driven by (non-Indigenous) researchers who ‘invited’ [82] the participation of Indigenous peoples, who were – at times – at arm’s length of the project phases. As suggested by the previously noted implications, participatory research with Indigenous peoples is everybody’s business. There are opportunities that scholars, practitioners, and Indigenous peoples might pursue if, as per the World Health Organization [1], ‘Health research involving Indigenous Peoples… [is] to be organized, designed and carried out in a manner that takes account of cultural differences, is based on mutual respect, and is beneficial and acceptable to both… [research institutions] and [Indigenous peoples]’.

## Additional file

Additional file 1: Publications that Met all Inclusion Criteria and included the Lexical (DOCX 222 kb)

## Data Availability

Not applicable.

## Abbreviations

CAQDAS: Computer assisted qualitative data analysis software
CBPR: Community-based participatory research
FN: First Nation
VRE: Video reflexive ethnography
