# Supplementary material for: What does it mean to conduct participatory research with Indigenous peoples? A lexical review
Source: BMC Public Health. 2019 Oct 29;19:1388. doi: 10.1186/s12889-019-7494-6 (PMC6819462; doi:10.1186/s12889-019-7494-6)
Supplement: Supplementary file 1 — Publications that Met all Inclusion Criteria and included the Lexical (DOCX 222 kb) [file 12889_2019_7494_MOESM1_ESM.docx]

# Additional file 1: Publications that Met all Inclusion Criteria and included the Lexical Review[[1-161](#_ENREF_1)]

1. Abbass-Dick J, Brolly M, Huizinga J, Newport A, Xie F, George S, Sterken E: **Designing an ehealth breastfeeding resource with Indigenous families using a participatory design**. *Journal of Transcultural Nursing* 2017, **29**(5):480-488.

2. Adams AK, LaRowe TL, Cronin KA, Prince RJ, Wubben DP, Parker T, Jobe JB: **The healthy children, strong families intervention: Design and community participation**. *Journal of Primary Prevention* 2012, **33**(4):175-185.

3. Adams K, Burns C, Liebzeit A, Ryschka J, Thorpe S, Browne J: **Use of participatory research and photo-voice to support urban Aboriginal healthy eating**. *Health Social & Care in the Community* 2012, **20**(5):497-505.

4. Allen J, Mohatt GV, Rasmus SM, Hazel KL, Thomas L, Lindley S: **The tools to understand: Community as co-researcher on culture-specific protective factors for Alaska Natives**. *Journal of prevention & intervention in the community* 2006, **32**(1-2):41-59.

5. Belone L, Orosco A, Damon E, Smith-McNeal W, Rae R, Sherpa ML, Myers OB, Omeh AO, Wallerstein N: **The piloting of a culturally centered American Indian family prevention program: A CBPR partnership between Mescalero Apache and the University of New Mexico**. *Public health reviews* 2017, **38**:1-13.

6. Bharadwaj L: **A framework for building research partnerships with first nations communities**. *Environmental health insights* 2014, **8**:15-25.

7. Boston P, Jordan S, MacNamara E, Kozolanka K, Bobbish-Rondeau E, Iserhoff H, Mianscum S, Mianscum-Trapper R, Mistacheesick I, Petawabano B *et al*: **Using participatory action research to understand the meanings Aboriginal Canadians attribute to the rising incidence of diabetes**. *Chronic diseases in Canada* 1997, **18**(1):5-12.

8. Boyer BB, Mohatt GV, Lardon C, Plaetke R, Luick BR, Hutchison SH, de Mayolo GA, Ruppert E, Bersamin A: **Building a community-based participatory research center to investigate obesity and diabetes in Alaska Natives**. *International journal of circumpolar health* 2005, **64**(3):281-290.

9. Brady M, MacKenzie-Taylor M: **Testing an Indigenous health resource: The participatory development process with 'the grog book'**. *Health Promotion Journal of Australia* 2002, **13**(3):247-249.

10. Brimblecombe J, Bailie R, van den Boogaard C, Wood B, Liberato SC, Ferguson M, Coveney J, Jaenke R, Ritchie J: **Feasibility of a novel participatory multi-sector continuous improvement approach to enhance food security in remote Indigenous Australian communities**. *SSM - population health* 2017, **3**:566-576.

11. Brussoni M, Olsen LL, Joshi P: **Aboriginal community-centered injury surveillance: A community-based participatory process evaluation**. *Prevention Science* 2012, **13**(2):107-117.

12. Bulman J, Hayes R: **Promoting Indigenous participation in health promotion education through community-based participatory research**. *Aboriginal and Islander Health Worker Journal* 2008, **32**(3):10-13.

13. Cargo M, Delormier T, Levesque L, Horn-Miller K, McComber A, Macaulay AC: **Can the democratic ideal of participatory research be achieved? An inside look at an academic-Indigenous community partnership**. *Health education research* 2008, **23**(5):904-914.

14. Castleden H, Garvin T: **Modifying photovoice for community-based participatory Indigenous research**. *Social Science & Medicine* 2008, **66**(6):1393-1405.

15. Castleden H, Morgan VS, Neimanis A: **Researchers' perspectives on collective/community co-authorship in community-based participatory Indigenous research**. *Journal of Empirical Research on Human Research Ethics* 2010, **5**(4):23-32.

16. Caxaj CS: **Indigenous storytelling and participatory action research: Allies toward decolonization? Reflections from the peoples' international health tribunal**. *Global qualitative nursing research* 2015, **2**:2333393615580764.

17. Charania NA, Tsuji LJ: **A community-based participatory approach and engagement process creates culturally appropriate and community informed pandemic plans after the 2009 H1N1 influenza pandemic: Remote and isolated first nations communities of sub-arctic Ontario, Canada**. *BMC public health* 2012, **12**:268.

18. Charania NA, Tsuji LJ: **Assessing the effectiveness and feasibility of implementing mitigation measures for an influenza pandemic in remote and isolated first nations communities: A qualitative community-based participatory research approach**. *Rural and remote health* 2013, **13**(4):2566.

19. Christopher S, Gidley AL, Letiecq B, Smith A, McCormick AK: **A cervical cancer community-based participatory research project in a Native American community**. *Health Education & Behavior* 2008, **35**(6):821-834.

20. Christopher S, Saha R, Lachapelle P, Jennings D, Colclough Y, Cooper C, Cummins C, Eggers MJ, Fourstar K, Harris K *et al*: **Applying Indigenous community-based participatory research principles to partnership development in health disparities research**. *Family & community health* 2011, **34**(3):246-255.

21. Cox A, Dudgeon P, Holland C, Kelly K, Scrine C, Walker R: **Using participatory action research to prevent suicide in Aboriginal and Torres Strait Islander communities**. *Australian journal of primary health* 2014, **20**(4):345-349.

22. Daley CM, Greiner KA, Nazir N, Daley SM, Solomon CL, Braiuca SL, Smith TE, Choi WS: **All nations breath of life: Using community-based participatory research to address health disparities in cigarette smoking among American Indians**. *Ethnicity & disease* 2010, **20**(4):334-338.

23. Dickson G: **Aboriginal grandmothers' experience with health promotion and participatory action research**. *Qualitative health research* 2000, **10**(2):188-213.

24. Dickson G, Green KL: **Participatory action research: Lessons learned with Aboriginal grandmothers**. *Health care for women international* 2001, **22**(5):471-482.

25. DiStefano A, Peters R, Tanjasiri SP, Quitugua L, Dimaculangan J, Hui B, Barrera-Ng A, Vunileva, Tui'one V, Takahashi L: **A community-based participatory research study of HIV and HPV vulnerabilities and prevention in two Pacific Islander communities: Ethical challenges and solutions**. *Journal of Empirical Research on Human Research Ethics* 2013, **8**(1):68-78.

26. Erick W, Mooney-Somers J, Akee A, Maher L: **Resilience to blood-borne and sexually transmitted infections: The development of a participatory action research project with young Aboriginal and Torres Strait Islander people in Townsville**. *Aboriginal and Islander Health Worker Journal* 2008, **32**(6):5-8.

27. Esler DM: **Participatory action research in Indigenous health**. *Australian family physician* 2008, **37**(6):457-459.

28. Farmer A, Gage J, Kirk R, Edgar T: **Applying community-based participatory research to create a diabetes prevention documentary with New Zealand Maori**. *Progress in Community Health Partnerships* 2016, **10**(3):383-390.

29. Ferrazzi P, Christie P, Jalovcic D, Tagalik S, Grogan A: **Reciprocal Inuit and Western research training: Facilitating research capacity and community agency in Arctic research partnerships**. *International journal of circumpolar health* 2018, **77**(1):1425581.

30. Fisher PA, Ball TJ: **The Indian family wellness project: An application of the tribal participatory research model**. *Prevention Science* 2002, **3**(3):235-240.

31. Fletcher G, Fredericks B, Adams K, Finlay S, Andy S, Briggs L, Hall R: **Having a yarn about smoking: Using action research to develop a 'no smoking' policy within an Aboriginal health organisation**. *Health policy (Amsterdam, Netherlands)* 2011, **103**(1):92-97.

32. Ford L, Klesch M: **'It won't matter soon, we'll all be dead': Endangered languages and action research. Wadeye Aboriginal languages project**. *Ngoonjook* 2003(23):27-43.

33. Ford T, Rasmus S, Allen J: **Being useful: Achieving Indigenous youth involvement in a community-based participatory research project in Alaska**. *International journal of circumpolar health* 2012, **71**(0):1-7.

34. Fuller J, Hermeston W, Passey M, Fallon T, Muyambi K: **Acceptability of participatory social network analysis for problem-solving in Australian Aboriginal health service partnerships**. *BMC health services research* 2012, **12**(152):1-11.

35. Gauld S, Smith S, Kendall MB: **Using participatory action research in community-based rehabilitation for people with acquired brain injury: From service provision to partnership with Aboriginal communities**. *Disability and rehabilitation* 2011, **33**(19-20):1901-1911.

36. Genuis SK, Willows N, Jardine CG: **Partnering with Indigenous student co-researchers: Improving research processes and outcomes**. *International journal of circumpolar health* 2015, **74**:27838.

37. Gittelsohn J, Roache C, Kratzmann M, Reid R, Ogina J, Sharma S: **Participatory research for chronic disease prevention in Inuit communities**. *American journal of health behavior* 2010, **34**(4):453-464.

38. Hecker R: **Participatory action research as a strategy for empowering Aboriginal health workers**. *Australian and New Zealand journal of public health* 1997, **21**(7):784-788.

39. Helm S, Okamoto SK, Medeiros H, Chin CI, Kawano KN, Po A-Kekuawela KO, Nebre LH, Sele FP: **Participatory drug prevention research in rural Hawai'i with native Hawaiian middle school students**. *Progress in Community Health Partnerships* 2008, **2**(4):307-313.

40. Henning J, Khin A, Hla T, Meers J: **Husbandry and trade of Indigenous chickens in Myanma r- Results of a participatory rural appraisal in the Yangon and the Mandalay divisions**. *Tropical animal health and production* 2006, **38**(7-8):611-618.

41. Heredia R: **Problems and possibilities of development work among tribals: A participatory evaluation of a voluntary agency in India**. *Community development journal* 1988, **23**(1):47-51.

42. Hickey SD, Maidment SJ, Heinemann KM, Roe YL, Kildea SV: **Participatory action research opens doors: Mentoring Indigenous researchers to improve midwifery in urban Australia**. *Women and Birth* 2017, **31**(4):263-268.

43. Hogan L, Garcia Bengoechea E, Salsberg J, Jacobs J, King M, Macaulay AC: **Using a participatory approach to the development of a school-based physical activity policy in an Indigenous community**. *Journal of School Health* 2014, **84**(12):786-792.

44. Horn K, McCracken L, Dino G, Brayboy M: **Applying community-based participatory research principles to the development of a smoking-cessation program for American Indian teens: "Telling our story"**. *Health Education & Behavior* 2008, **35**(1):44-69.

45. Jacklin K, Kinoshameg P: **Developing a participatory Aboriginal health research project: "Only if it's going to mean something"**. *Journal of Empirical Research on Human Research Ethics* 2008, **3**(2):53-67.

46. Jernigan V, Brokenleg I, Burkhart M, Magdalena C, Sibley C, Yepa K: **The implementation of a participatory manuscript development process with native American tribal awardees as part of the CDC communities putting prevention to work initiative: Challenges and opportunities**. *Preventive medicine* 2014, **67**(Supp. 1):S51-S57.

47. Jernigan VB: **Community-based participatory research with native American communities: The chronic disease self-management program**. *Health promotion practice* 2010, **11**(6):888-899.

48. Jernigan VB, Jacob T, Styne D: **The adaptation and implementation of a community-based participatory research curriculum to build tribal research capacity**. *American journal of public health* 2015, **105**(Supp. 3):S424-S432.

49. Jernigan VB, Salvatore AL, Styne DM, Winkleby M: **Addressing food insecurity in a native American reservation using community-based participatory research**. *Health education research* 2012, **27**(4):645-655.

50. Johansson P, Knox-Nicola P, Schmid K: **The Waponahki tribal health assessment: Successfully using CBPR to conduct a comprehensive and baseline health assessment of Waponahki tribal members**. *Journal of health care for the poor and underserved* 2015, **26**(3):889-907.

51. Jumper Thurman P, Allen J, Deters PB: **The circles of care evaluation: Doing participatory evaluation with American Indian and Alaska Native communities**. *American Indian and Alaska Native Mental Health Research* 2004, **11**(2):139-154.

52. Jumper-Reeves L, Dustman PA, Harthun ML, Kulis S, Brown EF: **American Indian cultures: How CBPR illuminated intertribal cultural elements fundamental to an adaptation effort**. *Prevention Science* 2014, **15**(4):547-556.

53. Kagawa-Singer M, Park Tanjasiri S, Lee SW, Foo MA, Ngoc Nguyen TU, Tran JH, Valdez A: **Breast and cervical cancer control among Pacific Islander and Southeast Asian Women: Participatory action research strategies for baseline data collection in California**. *Journal of Cancer Education* 2006, **21**(Supp. 1):S53-S60.

54. Katz JR, Martinez T, Paul R: **Community-based participatory research and American Indian/Alaska Native nurse practitioners: A partnership to promote adolescent health**. *Journal of the American Academy of Nurse Practitioners* 2011, **23**(6):298-304.

55. Kelley A, Medicine Bull LK, LaFranier G: **Participatory visual methods for American Indian communities and mental health conversations**. *American Indian and Alaska Native Mental Health Research* 2016, **23**(1):47-64.

56. Kerr S, Penney L, Barnes HM, McCreanor T: **Kaupapa Maori action research to improve heart disease services in Aotearoa, New Zealand**. *Ethnicity & health* 2010, **15**(1):15-31.

57. Kwan P, Sabado-Liwag M, Lee C, Lepule J, Pang V, Pike J, Santos L, Tanjasiri S, Toilolo T, Tui One May V *et al*: **Development of an online smoking cessation curriculum for Pacific Islanders: A community-based participatory research approach**. *Progress in Community Health Partnerships* 2017, **11**(3):263-274.

58. Kwan PP, Briand G, Lee C, Lepule JT, Pang JK, Sabado M, Sablan-Santos L, Schmidt-Vaivao D, Tanjasiri S, Tui'one V *et al*: **Use of a community-based participatory research approach to assess knowledge, attitudes, and beliefs on biospecimen research among Pacific Islanders**. *Health promotion practice* 2014, **15**(3):422-430.

59. Kyoon-Achan G, Lavoie J, Avery Kinew K, Phillips-Beck W, Ibrahim N, Sinclair S, Katz A: **Innovating for transformation in first nations health using community-based participatory research**. *Qualitative health research* 2018, **28**(7):1036-1049.

60. Langdon SE, Golden SL, Arnold EM, Maynor RF, Bryant A, Freeman VK, Bell RA: **Lessons learned from a community-based participatory research mental health promotion program for American Indian Youth**. *Health promotion practice* 2016, **17**(3):457-463.

61. Lonczak HS, Thomas LR, Donovan D, Austin L, Sigo RL, Lawrence N: **Navigating the tide together: Early collaboration between tribal and academic partners in a CBPR study**. *Pimatisiwin* 2013, **11**(3):395-409.

62. Lopez ED, Sharma DK, Mekiana D, Ctibor A: **Forging a new legacy of trust in research with Alaska Native college students using CBPR**. *International journal of circumpolar health* 2012, **71**:18475.

63. Macaulay AC, Delormier T, McComber AM, Cross EJ, Potvin LP, Paradis G, Kirby RL, Saad-Haddad C, Desrosiers S: **Participatory research with native community of Kahnawake creates innovative code of research ethics**. *Canadian Journal of Public Health* 1998, **89**(2):105-108.

64. Markus SF: **Photovoice for healthy relationships: Community-based participatory HIV prevention in a rural American Indian community**. *American Indian and Alaska Native Mental Health Research* 2012, **19**(1):102-123.

65. Matloub J, Creswell PD, Strickland R, Pierce K, Stephenson L, Waukau J, Kaur JS, Remington P: **Lessons learned from a community-based participatory research project to improve American Indian cancer surveillance**. *Progress in Community Health Partnerships* 2009, **3**(1):47-52.

66. Matsunaga DS, Enos R, Gotay CC, Banner RO, DeCambra H, Hammond OW, Hedlund N, Ilaban EK, Issell BF, Tsark JA: **Participatory research in a Native Hawaiian community. The Wai'anae cancer research project**. *Cancer* 1996, **78**(Supp. 7):1582-1586.

67. McCalman J, Tsey K, Baird B, Connolly B, Baird L, Jackson R: **'Bringing back respect': The role of participatory action research in transferring knowledge from an Aboriginal men's group to youth programs**. *Australasian psychiatry : bulletin of Royal Australian and New Zealand College of Psychiatrists* 2009, **17**(Supp. 1):S59-S63.

68. McClymont Peace D, Myers E: **Community-based participatory process - Climate change and health adaptation program for northern first nations and Inuit in Canada**. *International journal of circumpolar health* 2012, **71**:1-8.

69. McDonald EL, Bailie RS, Morris PS: **Participatory systems approach to health improvement in Australian Aboriginal children**. *Health promotion international* 2017, **32**(1):62-72.

70. McElfish PA, Narcisse MR, Long CR, Ayers BL, Hawley NL, Aitaoto N, Riklon S, Su LJ, Ima SZ, Wilmoth RO *et al*: **Leveraging community-based participatory research capacity to recruit Pacific Islanders into a genetics study**. *Journal of community genetics* 2017, **8**(4):283-291.

71. McMullin J, Bone M, Pang JK, Pang VK, McEligot A: **Native Hawaiian voices: Enhancing the role of cultural values in community based participatory research**. *Californian journal of health promotion* 2010, **8**:52-62.

72. Mendenhall TJ, Berge JM, Harper P, GreenCrow B, LittleWalker N, WhiteEagle S, BrownOwl S: **The family education diabetes series (FEDS): Community-based participatory research with a midwestern American Indian community**. *Nursing inquiry* 2010, **17**(4):359-372.

73. Miller A, Massey PD, Judd J, Kelly J, Durrheim DN, Clough AR, Speare R, Saggers S: **Using a participatory action research framework to listen to Aboriginal and Torres Strait Islander people in Australia about pandemic influenza**. *Rural and remote health* 2015, **15**(3):2923.

74. Minore B, Boone M, Katt M, Kinch P, Birch S: **Addressing the realities [correction of realties] of health care in northern Aboriginal communities through participatory action research**. *Journal of interprofessional care* 2004, **18**(4):360-368.

75. Mishra SI, Luce PH, Baquet CR: **Increasing pap smear utilization among Samoan women: Results from a community based participatory randomized trial**. *Journal of health care for the poor and underserved* 2009, **20**(Supp. 2):85-101.

76. Mohammed SA, Walters KL, Lamarr J, Evans-Campbell T, Fryberg S: **Finding middle ground: Negotiating university and tribal community interests in community-based participatory research**. *Nursing inquiry* 2012, **19**(2):116-127.

77. Mohatt GV, Hazel KL, Allen J, Stachelrodt M, Hensel C, Fath R: **Unheard Alaska: Culturally anchored participatory action research on sobriety with Alaska Natives**. *American journal of community psychology* 2004, **33**(3-4):263-273.

78. Mohatt GV, Plaetke R, Klejka J, Luick B, Lardon C, Bersamin A, Hopkins S, Dondanville M, Herron J, Boyer B: **The center for Alaska Native health research study: A community-based participatory research study of obesity and chronic disease-related protective and risk factors**. *International journal of circumpolar health* 2007, **66**(1):8-18.

79. Mohindra K, Narayana D, Harikrishnadas C, Anushreedha S, Haddad S: **Paniya voices: A participatory poverty and health assessment among a marginalized south Indian tribal population**. *BMC public health* 2010, **10**:149.

80. Mohindra KS, Narayana D, Haddad S: **"My story is like a goat tied to a hook." Views from a marginalized tribal group in Kerala (India) on the consequences of falling ill: A participatory poverty and health assessment**. *Journal of epidemiology and community health* 2010, **64**(6):488-494.

81. Mokuau N, Browne CV, Braun KL, Choy LB: **Using a community-based participatory approach to create a resource center for Native Hawaiian elders**. *Education for Health* 2008, **21**(3):174.

82. Mooney-Somers J, Erick W, Scott R, Akee A, Kaldor J, Maher L: **Enhancing Aboriginal and Torres Strait Islander young people's resilience to blood-borne and sexually transmitted infections: Findings from a community-based participatory research project**. *Health Promotion Journal of Australia* 2009, **20**(3):195-201.

83. Mooney-Somers J, Maher L: **The Indigenous resiliency project: A worked example of community-based participatory research**. *New South Wales public health bulletin* 2009, **20**(7-8):112-118.

84. Moran MF: **An evaluation of participatory planning at Mapoon Aboriginal community: Opportunities for inclusive local governance**. *Australian Aboriginal Studies* 2003(2):72-84.

85. Morris M: **Inuit involvement in developing a participatory action research project on youth, violence prevention, and health promotion**. *Inuit Studies* 2016, **40**(1):105-125.

86. Mullany B, Barlow A, Neault N, Billy T, Jones T, Tortice I, Lorenzo S, Powers J, Lake K, Reid R *et al*: **The family spirit trial for American Indian teen mothers and their children: CBPR rationale, design, methods and baseline characteristics**. *Prevention Science* 2012, **13**(5):504-518.

87. Munns A, Toye C, Hegney D, Kickett M, Marriott R, Walker R: **Peer-led Aboriginal parent support: Program development for vulnerable populations with participatory action research**. *Contemporary nurse* 2017, **53**(5):558-575.

88. Munns A, Toye C, Hegney D, Kickett M, Marriott R, Walker R: **Aboriginal parent support: A partnership approach**. *Journal of clinical nursing* 2018, **27**(3-4):e437-e450.

89. Munro A, Shakeshaft A, Clifford A: **The development of a healing model of care for an Indigenous drug and alcohol residential rehabilitation service: A community-based participatory research approach**. *Health & justice* 2017, **5**(1):12.

90. Nilson C, Kearing-Salmon KA, Morrison P, Fetherston C: **An ethnographic action research study to investigate the experiences of Bindjareb women participating in the cooking and nutrition component of an Aboriginal health promotion programme in regional Western Australia**. *Public health nutrition* 2015, **18**(18):3394-3405.

91. Noe TD, Manson SM, Croy C, McGough H, Henderson JA, Buchwald DS: **The influence of community-based participatory research principles on the likelihood of participation in health research in American Indian communities**. *Ethnicity & disease* 2007, **17**(Supp. 1):S6-14.

92. Oster RT, Bruno G, Montour M, Roasting M, Lightning R, Rain P, Graham B, Mayan MJ, Toth EL, Bell RC: **Kikiskawawasow - Prenatal healthcare provider perceptions of effective care for first nations women: An ethnographic community-based participatory research study**. *BMC pregnancy and childbirth* 2016, **16**(1):216.

93. Pahwa P, Abonyi S, Karunanayake C, Rennie DC, Janzen B, Kirychuk S, Lawson JA, Katapally T, McMullin K, Seeseequasis J *et al*: **A community-based participatory research methodology to address, redress, and reassess disparities in respiratory health among first nations**. *BMC Resesearch Notes* 2015, **8**:199.

94. Panapasa S, Jackson J, Caldwell C, Heeringa S, McNally J, Williams D, Coral D, Taumoepeau L, Young L, Young S *et al*: **Community-based participatory research approach to evidence-based research: Lessons from the Pacific Islander American health study**. *Progress in Community Health Partnerships* 2012, **6**(1):53-58.

95. Pelcastre-Villafuerte BE, Meneses-Navarro S, Ruelas-Gonzalez MG, Reyes-Morales H, Amaya-Castellanos A, Taboada A: **Aging in rural, Indigenous communities: An intercultural and participatory healthcare approach in Mexico**. *Ethnicity & health* 2017, **22**(6):610-630.

96. Perry C, Hoffman B: **Assessing tribal youth physical activity and programming using a community-based participatory research approach**. *Public Health Nursing* 2010, **27**(2):104-114.

97. Petersen DM, Minkler M, Vasquez VB, Kegler MC, Malcoe LH, Whitecrow S: **Using community-based participatory research to shape policy and prevent lead exposure among Native American children**. *Progress in Community Health Partnerships* 2007, **1**(3):249-256.

98. Puertas B, Schlesser M: **Assessing community health among Indigenous populations in Ecuador with a participatory approach: Implications for health reform**. *Journal of community health* 2001, **26**(2):133-147.

99. Quigley D, Handy D, Goble R, Sanchez V, George P: **Participatory research strategies in nuclear risk management for native communities**. *Journal of health communication* 2000, **5**(4):305-331.

100. Rasmus SM: **Indigenizing CBPR: Evaluation of a community-based and participatory research process implementation of the Elluam Tungiinun (towards wellness) program in Alaska**. *American journal of community psychology* 2014, **54**(1-2):170-179.

101. Ratima MM, Fox C, Fox B, Te Karu H, Gemmell T, Slater T, D'Souza WJ, Pearce NE: **Long-term benefits for Maori of an asthma self-management program in a Maori community which takes a partnership approach**. *Australian and New Zealand journal of public health* 1999, **23**(6):601-605.

102. Redwood D, Lanier A, Kemberling M, Klejka J, Sylvester I, Lundgren K: **Community-based participatory research in a large cohort study of chronic diseases among Alaska native adults**. *Progress in Community Health Partnerships* 2010, **4**(4):325-330.

103. Richards J, Mousseau A: **Community-based participatory research to improve preconception health among Northern Plains American Indian adolescent women**. *American Indian and Alaska native Mental Health Research* 2012, **19**(1):154-185.

104. Richmond LS, Peterson DJ, Betts SC: **The evolution of an evaluation: A case study using the tribal participatory research model**. *Health promotion practice* 2008, **9**(4):368-377.

105. Ritchie SD, Jo Wabano M, Beardy J, Curran J, Orkin A, VanderBurgh D, Young NL: **Community-based participatory research with Indigenous communities: The proximity paradox**. *Health & place* 2013, **24**:183-189.

106. SantoDomingo AF, Castro-Diaz L, Gonzalez-Uribe C: **Ecosystem research experience with two Indigenous communities of Colombia: The ecohealth calendar as a participatory and innovative methodological tool**. *EcoHealth* 2016, **13**(4):687-697.

107. Schurer JM, Phipps K, Okemow C, Beatch H, Jenkins E: **Stabilizing dog populations and improving animal and public health through a participatory approach in Indigenous communities**. *Zoonoses and public health* 2015, **62**(6):445-455.

108. Scott S, D'Silva J, Hernandez C, Villaluz NT, Martinez J, Matter C: **The tribal tobacco education and policy initiative: Findings from a collaborative, participatory evaluation**. *Health promotion practice* 2017, **18**(4):545-553.

109. Sherwood J, Kendall S: **Reframing spaces by building relationships: Community collaborative participatory action research with Aboriginal mothers in prison**. *Contemporary nurse* 2013, **46**(1):83-94.

110. Strickland CJ: **Challenges in community-based participatory research implementation: Experiences in cancer prevention with pacific northwest American Indian tribes**. *Cancer control : journal of the Moffitt Cancer Center* 2006, **13**(3):230-236.

111. Subrahmanian K, Petereit DG, Kanekar S, Burhansstipanov L, Esmond S, Miner R, Spotted Tail C, Guadagnolo BA: **Community-based participatory development, implementation, and evaluation of a cancer screening educational intervention among American Indians in the northern plains**. *Journal of Cancer Education* 2011, **26**(3):530-539.

112. Tanjasiri SP, Weiss JW, Santos L, Flores P, Flores P, Lacsamana JD, Paige C, Mouttapa M, Quitugua L, Taito P *et al*: **CBPR-informed recruitment and retention adaptations in a randomized study of pap testing among Pacific Islanders in Southern California**. *Progress in Community Health Partnerships* 2015, **9**(3):389-396.

113. Tanjasiri SP, Wiersma L, Briand G, Faletau V, Lepule J, Nacpil L, Eichenauer J: **Balancing community and university aims in community-based participatory research: a Pacific Islander youth study**. *Progress in Community Health Partnerships* 2011, **5**(1):19-25.

114. Thomas LR, Donovan DM, Sigo RL: **Identifying community needs and resources in a native community: A research partnership in the pacific Northwest**. *International journal of mental health and addiction* 2010, **8**(2):362-373.

115. Thomas LR, Rosa C, Forcehimes A, Donovan DM: **Research partnerships between academic institutions and American Indian and Alaska Native tribes and organizations: Effective strategies and lessons learned in a multisite CTN study**. *American Journal of Drug and Alcohol Abuse* 2011, **37**(5):333-338.

116. Tilburt JC, James KM, Koller K, Lanier AP, Hall IJ, Smith JL, Ekwueme DU, Nicometo AM, Petersen WO: **Assessing follow-up care after prostate-specific antigen elevation in American Indian / Alaska Native Men: A partnership approach**. *Progress in Community Health Partnerships* 2013, **7**(2):153-161.

117. Tiwari T, Sharma T, Harper M, Zacher T, Roan R, George C, Swyers E, Toledo N, Batliner T, Braun PA *et al*: **Community based participatory research to reduce oral health disparities in American Indian children**. *Journal of family medicine* 2015, **2**(3):1028.

118. Townsend CK, Dillard A, Hosoda KK, Maskarinec GG, Maunakea AK, Yoshimura SR, Hughes C, Palakiko DM, Kehauoha BP, Kaholokula JK: **Community-based participatory research integrates behavioral and biological research to achieve health equity for native Hawaiians**. *International journal of environmental research and public health* 2015, **13**(1):ijerph13010004.

119. Trotter RTn, Laurila K, Alberts D, Huenneke LF: **A diagnostic evaluation model for complex research partnerships with community engagement: The partnership for Native American cancer prevention (NACP) model**. *Evaluation and program planning* 2015, **48**:10-20.

120. Tsark JA: **A participatory research approach to address data needs in tobacco use among Native Hawaiians**. *Asian American and Pacific Islander journal of health* 2001, **9**(1):40-48.

121. Tsey K, Patterson D, Whiteside M, Baird L, Baird B: **Indigenous men taking their rightful place in society? A preliminary analysis of a participatory action research process with Yarrabah men's health group**. *Australian Journal of Rural Health* 2002, **10**(6):278-284.

122. Ulturgasheva O, Wexler L, Kral M, Allen J, Mohatt GV, Nystad K: **Navigating international, interdisciplinary, and indigenous collaborative inquiry: Phase 1 in the circumpolar Indigenous Pathways to adulthood project**. *Journal of community engagement and scholarship* 2011, **4**(1):50-59.

123. VanderBurgh D, Jamieson R, Beardy J, Ritchie SD, Orkin A: **Community-based first aid: A program report on the intersection of community-based participatory research and first aid education in a remote Canadian Aboriginal community**. *Rural and remote health* 2014, **14**:2537.

124. Varcoe C, Brown H, Calam B, Harvey T, Tallio M: **Help bring back the celebration of life: A community-based participatory study of rural Aboriginal women's maternity experiences and outcomes**. *BMC pregnancy and childbirth* 2013, **13**:26.

125. Vergara-Asenjo G, Mateo-Vega J, Alvarado A, Potvin C: **A participatory approach to elucidate the consequences of land invasions on REDD+ initiatives: A case study with Indigenous communities in Panama**. *PloS one* 2017, **12**(12):e0189463.

126. Verney SP, Avila M, Espinosa PR, Cholka CB, Benson JG, Baloo A, Pozernick CD: **Culturally sensitive assessments as a strength-based approach to wellness in Native communities: A community-based participatory research project**. *American Indian and Alaska Native Mental Health Research* 2016, **23**(3):271-292.

127. Waldner CL, Alimezelli HT, McLeod L, Zagozewski R, Bradford LE, Bharadwaj LA: **Self-reported effects of water on health in first nations communities in Saskatchewan, Canada: Results from community-based participatory research**. *Environmental health insights* 2017, **11**:1178630217690193.

128. Whitewater S, Reinschmidt KM, Kahn C, Attakai A, Teufel-Shone NI: **Flexible roles for American Indian Elders in community-based participatory research**. *Preventing chronic disease* 2016, **13**:E72.

129. Whitty-Rogers J, Caine V, Cameron B: **Aboriginal women's experiences with gestational diabetes mellitus: A participatory study with Mi'kmaq women in Canada**. *Advances in Nursing Science* 2016, **39**(2):181-198.

130. Wood L: **'Every teacher is a researcher!': Creating indigenous epistemologies and practices for HIV prevention through values-based action research**. *SAHARA J : journal of Social Aspects of HIV/AIDS Research Alliance* 2012, **9**(Supp. 1):S19-S27.

131. Young C, Tong A, Sherriff S, Kalucy D, Fernando P, Muthayya S, Craig JC: **Building better research partnerships by understanding how Aboriginal health communities perceive and use data: A semistructured interview study**. *BMJ open* 2016, **6**(4):e010792.

132. Zagozewski R, Judd-Henrey I, Nilson S, Bharadwaj L: **Perspectives on past and present waste disposal practices: A community-based participatory research project in three Saskatchewan first nations communities**. *Environmental health insights* 2011, **5**:9-20.

133. Bat M, Fasoli L: **Action research as a both-ways curriculum development approach: Supporting self-determination in the remote Indigenous child care workforce in the Northern Territory of Australia**. *Action Research* 2013, **11**(1):52-72.

134. Blodgett AT, Schinke RJ, Peltier D, Fisher LA, Watson J, Wabano MJ: **May the circle be unbroken: The research recommendations of Aboriginal community members engaged in participatory action research with university academics**. *Journal of Sport & Social Issues* 2011, **35**(3):264-283.

135. Blue Bird Jernigan V, Brokenleg IS, Burkhart M, Magdalena C, Sibley C, Yepa K: **The implementation of a participatory manuscript development process with Native American tribal awardees as part of the CDC communities putting prevention to work initiative: Challenges and opportunities**. *Preventive medicine* 2014, **67**(Supp. 1):S51-S57.

136. Blue Bird Jernigan V, Salvatore AL, Styne DM, Winkleby M: **Addressing food insecurity in a Native American reservation using community-based participatory research**. *Health education research* 2012, **27**(4):645-655.

137. Bulman J, Hayes R: **Mibbinbah: Empowering Indigenous males through participatory action research into health**. *Update Newsletter of the Australian Health Promotion Association* 2008:7.

138. Datta R, Khyang NU, Khyang HKP, Kheyang HAP, Khyang MC, Chapola J: **Participatory action research and researcher's responsibilities: An experience with an Indigenous community**. *International Journal of Social Research Methodology: Theory & Practice* 2015, **18**(6):581-599.

139. Edmunds DS, Shelby R, James A, Steele L, Baker M, Perez YV, TallBear K: **Tribal housing, codesign, and cultural sovereignty**. *Science, Technology & Human Values* 2013, **38**(6):801-828.

140. Fitzpatrick E, Martiniuk A, D'Antoine H, Oscar J, Carter M, Lawford T, Macdonald G, Hunter C, Elliott E: **Yarning with remote Aboriginal communities about seeking consent for research, culturally respectful community engagement and genuine research partnerships**. *Internal Medicine Journal* 2017, **47**:22-22.

141. Hayhurst LM, Giles AR, Radforth WM: **'I want to come here to prove them wrong': Using a post-colonial feminist participatory action research (PFPAR) approach to studying sport, gender and development programmes for urban Indigenous young women**. *Sport in Society* 2015, **18**(8):952-967.

142. Herlihy PH: **Participatory research mapping of Indigenous lands in Darién, Panama**. *Human Organization* 2003, **62**(4):315-331.

143. Holkup PA, Swaney G, Salois EM, Belcourt A: **CBPR: Grief and historical trauma within a tribal community**. *Communicating Nursing Research* 2011, **44**:473-473.

144. Johnson SR, Finifrock DA, Marshall CA, Jaakola J, Setterquist J, Burross HL, Hodge FS: **Cancer, employment, and American Indians: A participatory action research pilot study**. *Rehabilitation Counseling Bulletin* 2011, **54**(3):175-180.

145. Josif C, Barclay L, Bar-Zeev S, Kildea S, Brittin M: **How participatory action research supported improvements to the postnatal discharge summary system used for remote dwelling Aboriginal mothers and infants in the top end of Australia**. *Action Research* 2012, **10**(4):387-405.

146. McHugh T-LF, Kowalski KC: **'A new view of body image': A school-based participatory action research project with young Aboriginal women**. *Action Research* 2011, **9**(3):220-241.

147. McKenzie B, Seidl E, Bone N: **Child and family services standards in first nations: An action research project**. *Child Welfare* 1995, **74**(3):633-653.

148. Mills J, Felton-Busch C, Park T, Maza K, Mills F, Ghee M, Hitchins M, Chamberlain-Salaun J, Neuendorf N: **Supporting Australian Torres Strait Islander and Aboriginal nursing students using mentoring circles: An action research study**. *Higher Education Research & Development* 2014, **33**(6):1136-1149.

149. Moffitt P, Nurcombe B: **Action research: A pre-school for rural Aborigines and Europeans**. *Australian Psychologist* 1970, **5**(3):243-248.

150. Mooney-Somers J, Olsen A, Erick W, Scott R, Akee A, Maher L: **Young Indigenous Australians' sexually transmitted infection prevention practices: A community-based participatory research project**. *Journal of Community & Applied Social Psychology* 2012, **22**(6):519-532.

151. Peterson SS: **Developing a play-based communication assessment through collaborative action research with teachers in northern Canadian Indigenous communities**. *Literacy* 2017, **51**(1):36-43.

152. Riecken T, Conibear F, Michel C, Lyall J, Scott T, Tanaka M, Stewart S, Riecken J, Strong-Wilson T: **Resistance through re-presenting culture: Aboriginal student filmmakers and a participatory action research project on health and wellness**. *Canadian Journal of Education* 2006, **29**(1):265-286.

153. Roberts EB, Jette SL: **Implementing participatory research with an urban American Indian community: Lessons learned**. *Health Education Journal* 2016, **75**(2):158-169.

154. Schiller U, de Wet G: **Communication, Indigenous culture and participatory decision making amongst foster adolescents**. *Qualitative Social Work* 2018, **17**(2):236-251.

155. Schinke RJ, McGannon KR, Watson J, Busanich R: **Moving toward trust and partnership: An example of sport-related community-based participatory action research with Aboriginal people and mainstream academics**. *Journal of Aggression, Conflict and Peace Research* 2013, **5**(4):201-210.

156. Smith DA, Herlihy PH, Ramos Viera A, Kelly JH, Hilburn AM, Aguilar Robledo M, Dobson JE: **Using participatory research mapping and GIS to explore local geographic knowledge of Indigenous landscapes in Mexico**. *American Geographical Society's Focus on Geography* 2012, **55**(4):119-124.

157. Sun J, Buys N: **Participatory community singing program to enhance quality of life and social and emotional well-being in Aboriginal and Torres Strait Islander Australians with chronic diseases**. *International Journal on Disability and Human Development* 2013, **12**(3):317-323.

158. Sun J, Buys NJ: **Improving Aboriginal and Torres Strait Islander Australians' well-being using participatory community singing approach**. *International Journal on Disability and Human Development* 2013, **12**(3):305-316.

159. Thurman PJ, Allen J, Deters PB: **The circles of care: Doing participatory evaluation with American Indian and Alaska Native communities**. *American Indian and Alaska Native Mental Health Research* 2004, **11**(2):139-154.

160. Tuck E: **Re-visioning action: Participatory action research and Indigenous theories of change**. *The Urban Review* 2009, **41**(1):47-65.

161. Veroff S: **Participatory art research: Transcending barriers and creating knowledge and connection with young Inuit adults**. *American Behavioral Scientist* 2002, **45**(8):1273-1287.
